# Supplementary material for: In Vivo Microelectrode Arrays for Detecting Multi-Region Epileptic Activities in the Hippocampus in the Latent Period of Rat Model of Temporal Lobe Epilepsy
Source: Micromachines (Basel). 2021 Jun 3;12(6):659. doi: 10.3390/mi12060659 (PMC8228658; doi:10.3390/mi12060659)
Supplement: Supplementary file 1 [file micromachines-12-00659-s001.zip › micromachines-1233907-supplementary.pdf]

micromachines

Supplementary Materials

In Vivo Microelectrode Arrays for Detecting Multi-Region Epileptic Activities in the Hippocampus in the Latent Period of Rat Model of Temporal Lobe Epilepsy

Yuchuan Dai [1,2], Yilin Song [1,2], Jingyu Xie [1,2], Shengwei Xu [1,2], Xinrong Li [1,2], Enhui He [1,2], Huabing Yin [3] and Xinxia Cai [1,2,*]

1 State Key Laboratory of Transducer Technology, Aerospace Information Research Institute, Chinese Academy of Sciences, Beijing 100190, China; daiyuchuan18@mails.ucas.edu.cn (Y.D.); ylsong@mail.ie.ac.cn (Y.S.); xiejingyu16@mails.ucas.ac.cn (J.X.); swxu@mail.ie.ac.cn (S.X.); lxr8118@126.com (X.L.); heenhui17@mails.ucas.ac.cn (E.H.).
2 School of Electronic, Electrical and Communication Engineering, University of Chinese Academy of Sciences, Beijing 100049, China
3 Division of Biomedical Engineering, University of Glasgow Room 626, Rankine Building, Oakfield Avenue, Glasgow G12 8LT, UK; Huabing.Yin@glasgow.ac.uk (H.Y.).
* Correspondence: xxcai@mail.ie.ac.cn; Tel.: +86-10-58887193

Citation: Dai, Y.; Song, Y.; Xie, J.; Xu, S.; Li, X.; He, E.; Yin, H.; Cai, X. In Vivo Microelectrode Arrays for Detecting Multi-Region Epileptic Activities in the Hippocampus in the Latent Period of Rat Model of Temporal Lobe Epilepsy. *Micromachines* **2021**, *12*, 659. https://doi.org/10.3390/mi12060659

Academic Editor: Aiqun Liu

Received: 8 May 2021
Accepted: 30 May 2021
Published: 3 June 2021

Publisher's Note: MDPI stays neutral with regard to jurisdictional claims in published maps and institutional affiliations.

Copyright: © 2021 by the authors. Licensee MDPI, Basel, Switzerland. This article is an open access article distributed under the terms and conditions of the Creative Commons Attribution (CC BY) license (http://creativecommons.org/licenses/by/4.0/).

Supplementary figures

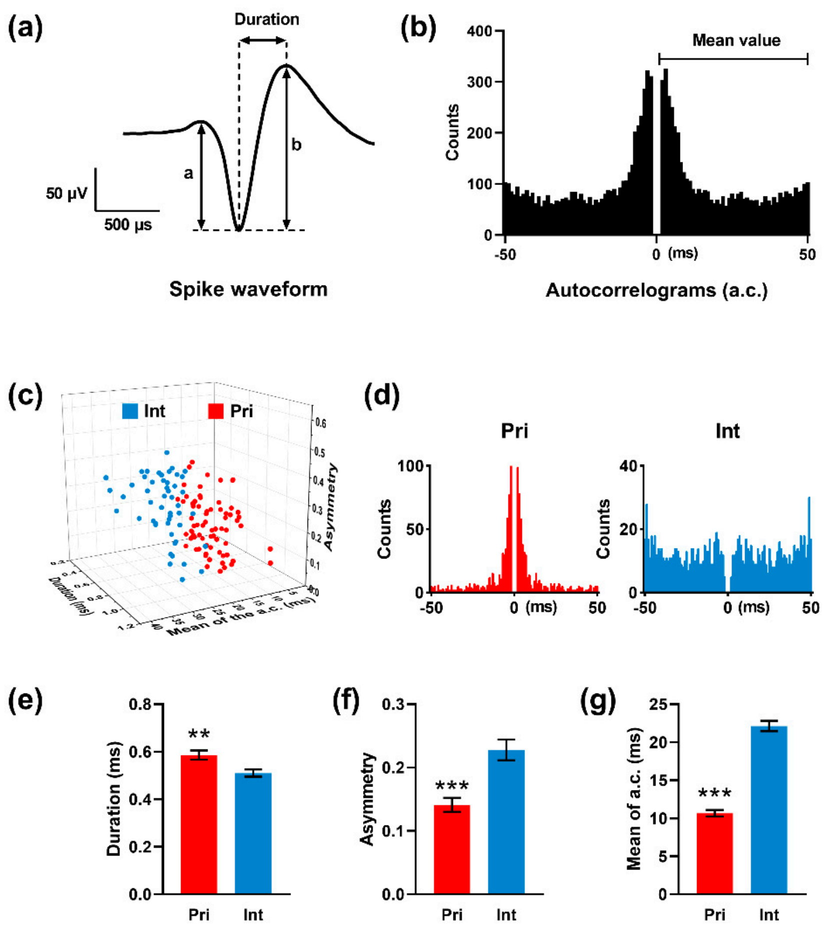

Figure S1. Classification of principal cells and interneurons. (**a**) Duration of spike waveform was the time interval between peak and valley. "a" represents the amplitude of pre-peak while "b" represents that of the post-peak. Asymmetry = (b−a)/(a + b). (**b**) Mean value of autocorrelograms in 50 ms was the third parameter for classification. (**c**) K-means clustering with duration, asymmetry and the mean of autocorrelograms. (**d**) In the present study, the autocorrelograms of principal cells showed peaks at 2–5 ms, whereas those of interneurons distributed with no apparent peaks. (**e**) The spike durations of principal cells and interneurons were 0.58 ± 0.16 ms and 0.50 ± 0.11 ms, respectively. (**f**) The asymmetry of principal cells (0.14 ± 0.09) was lower than that of interneurons (0.23 ± 0.12). (**g**) The mean value of autocorrelograms of principal cells (10.65 ± 3.58 ms) in 50 ms was significantly shorter than that of interneurons (22.13 ± 4.84 ms). The results indicated that the neural spikes were successfully divided into two groups corresponding to principal cells and interneurons. $^{**}P < 0.01$ and $^{***}P < 0.001$, unpaired *t*-test, n = 68 and 53 for pri and int respectively. Pri, principal cells. Int, interneurons.
